# Supplementary material for: Analysis of chi angle distributions in free amino acids via multiplet fitting of proton scalar couplings
Source: Magn Reson (Gott). 2024 Aug 19;5(2):103–20. doi: 10.5194/mr-5-103-2024 (PMC11570886; doi:10.5194/mr-5-103-2024)
Supplement: The supplement related to this article is available online at: https://doi.org/10.5194/mr-5-103-2024-supplement. [file mr-5-103-2024-supplement.zip › Syed2024/data/fit1d_fitnmr_output.tar.gz!/fit1d/his/500/bmse000039_simulation_1_nmredata/1H/resonances_1d.pdf]

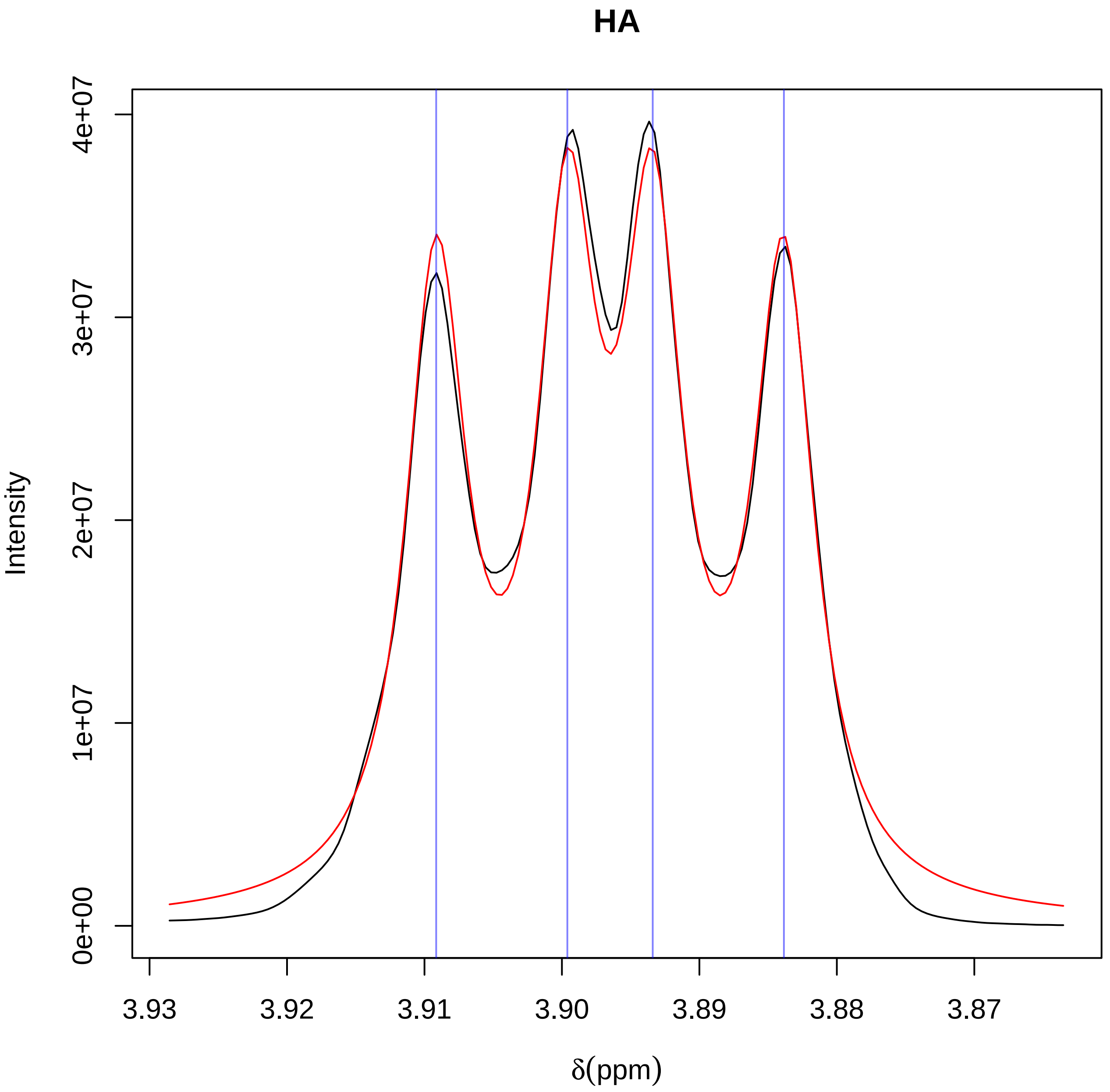

HA
Intensity
0e+00
1e+07
2e+07
3e+07
4e+07
3.93
3.92
3.91
3.90
3.89
3.88
3.87
δ(ppm)

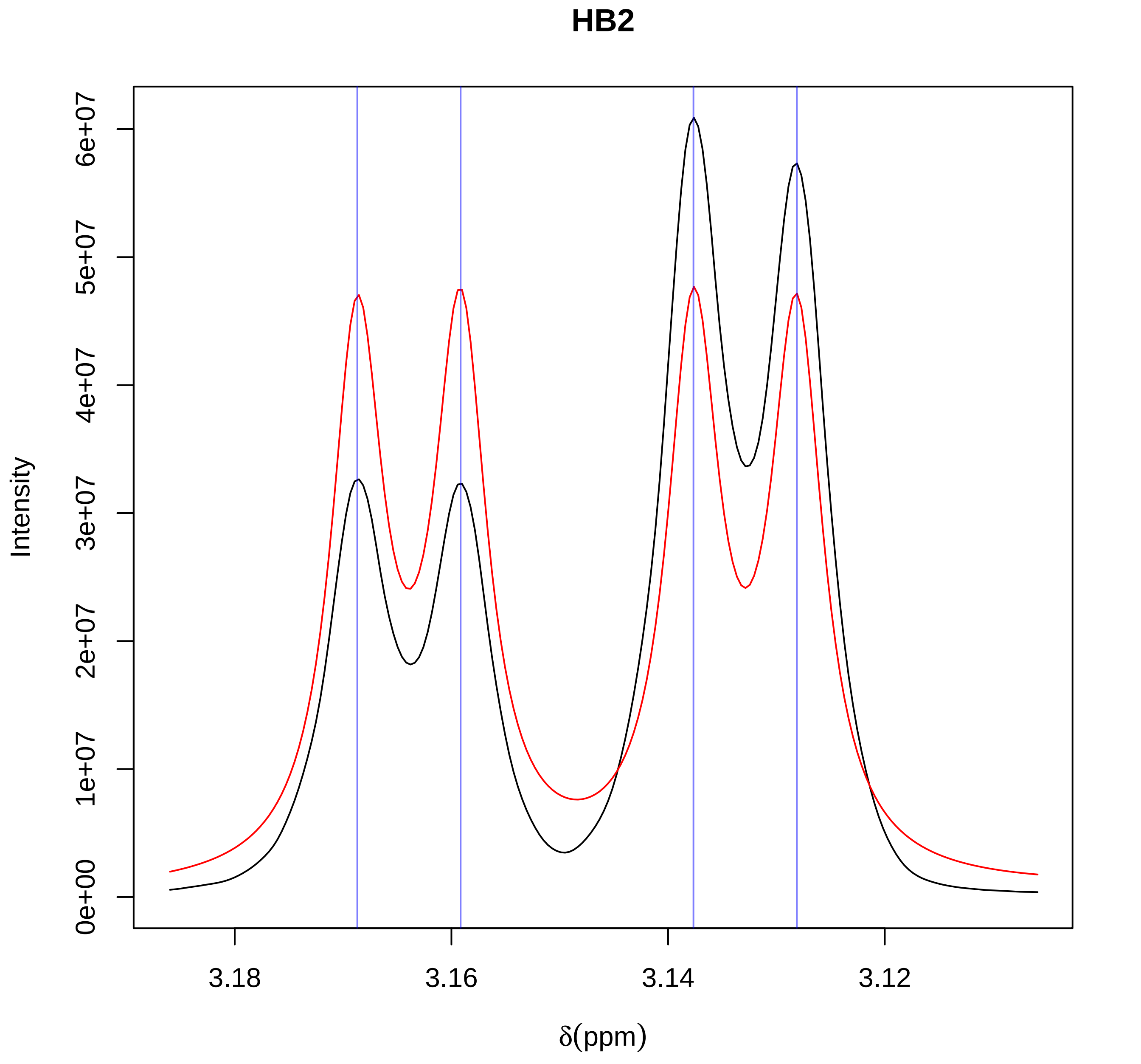

HB2
Intensity
6e+07
5e+07
4e+07
3e+07
2e+07
1e+07
0e+00
3.18
3.16
3.14
3.12
δ(ppm)

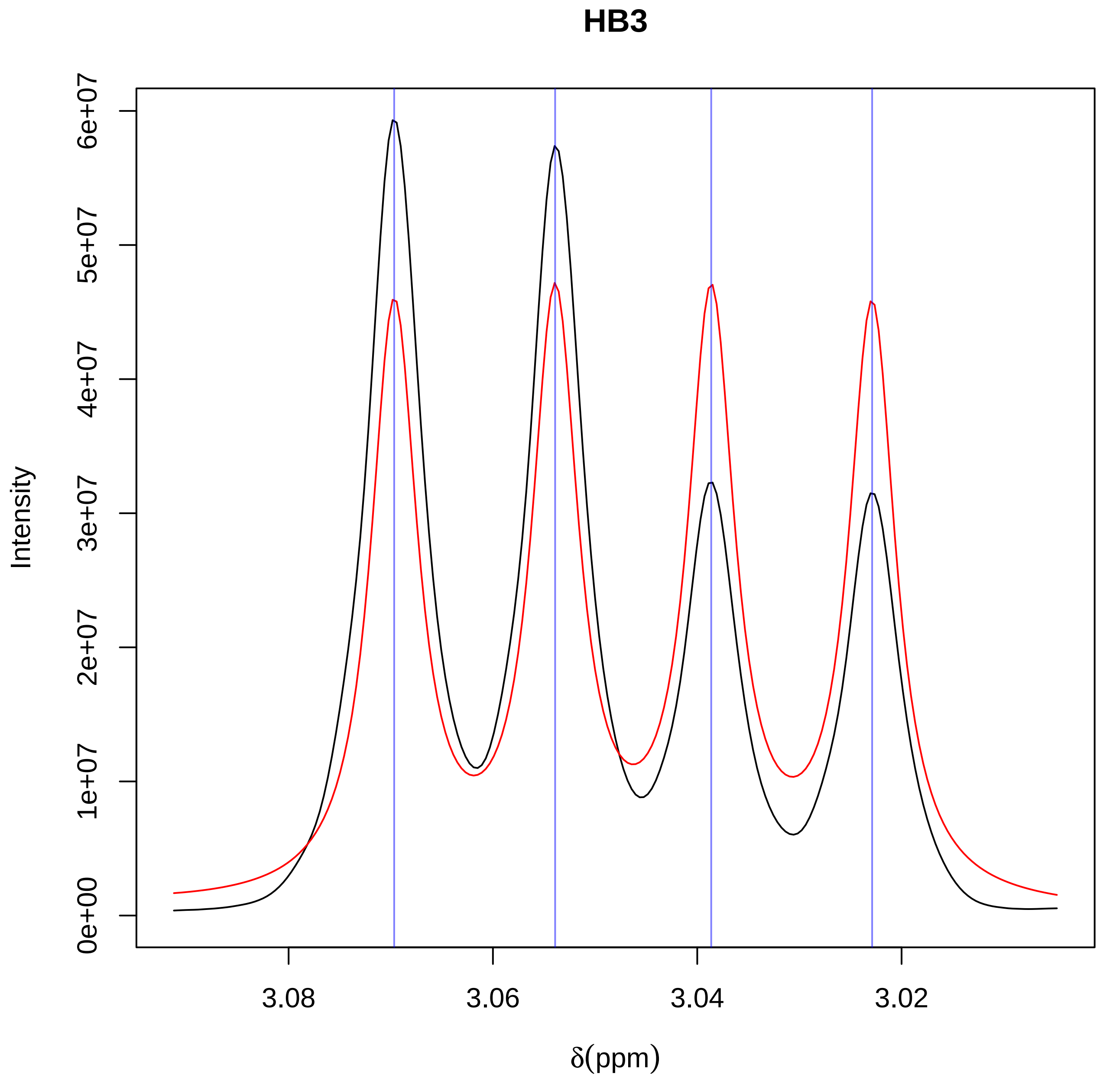
HB3
Intensity
0e+00
1e+07
2e+07
3e+07
4e+07
5e+07
6e+07
3.08
3.06
3.04
3.02
δ(ppm)

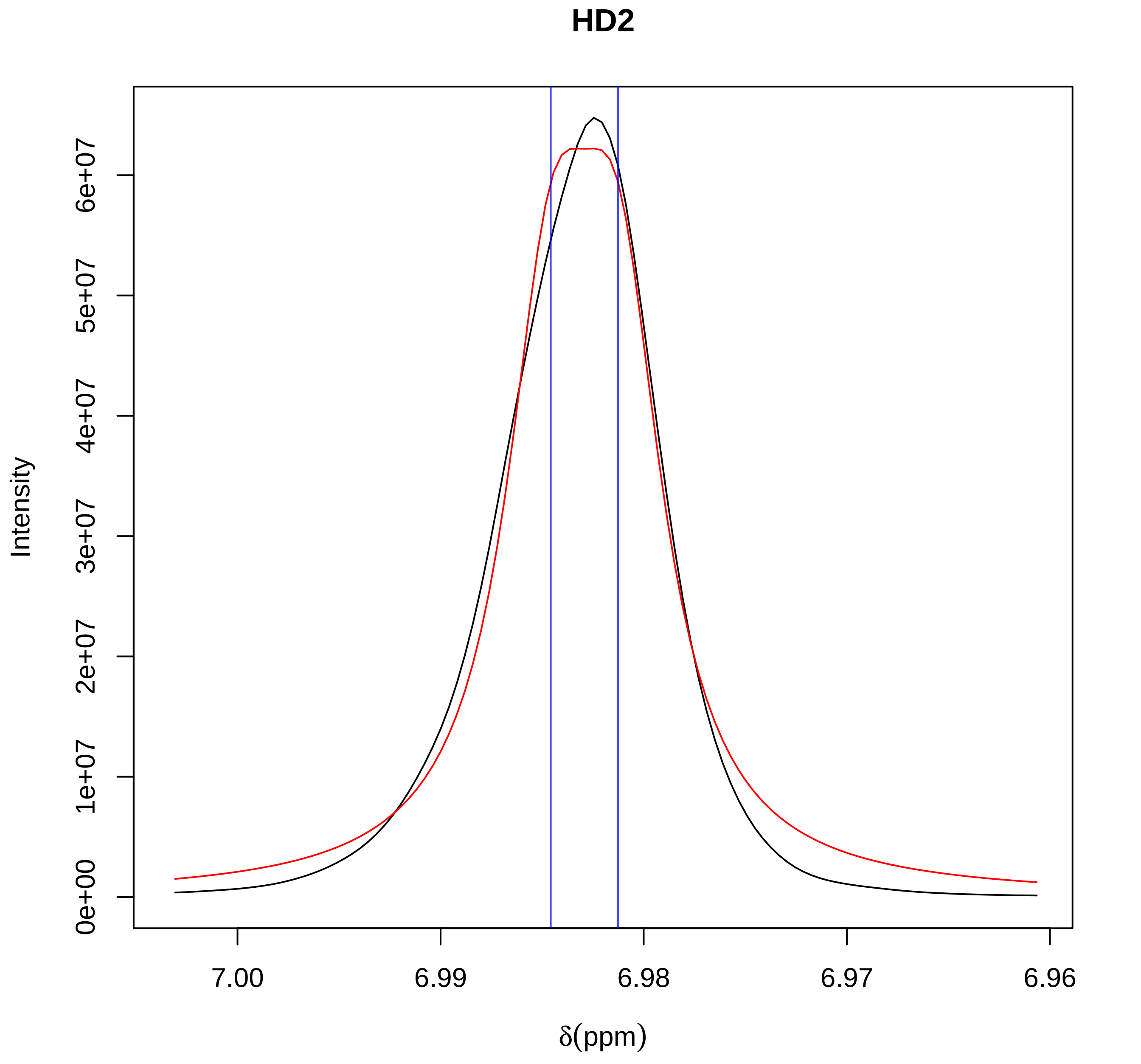
HD2
Intensity
0e+00
1e+07
2e+07
3e+07
4e+07
5e+07
6e+07
7.00
6.99
6.98
6.97
6.96
δ(ppm)

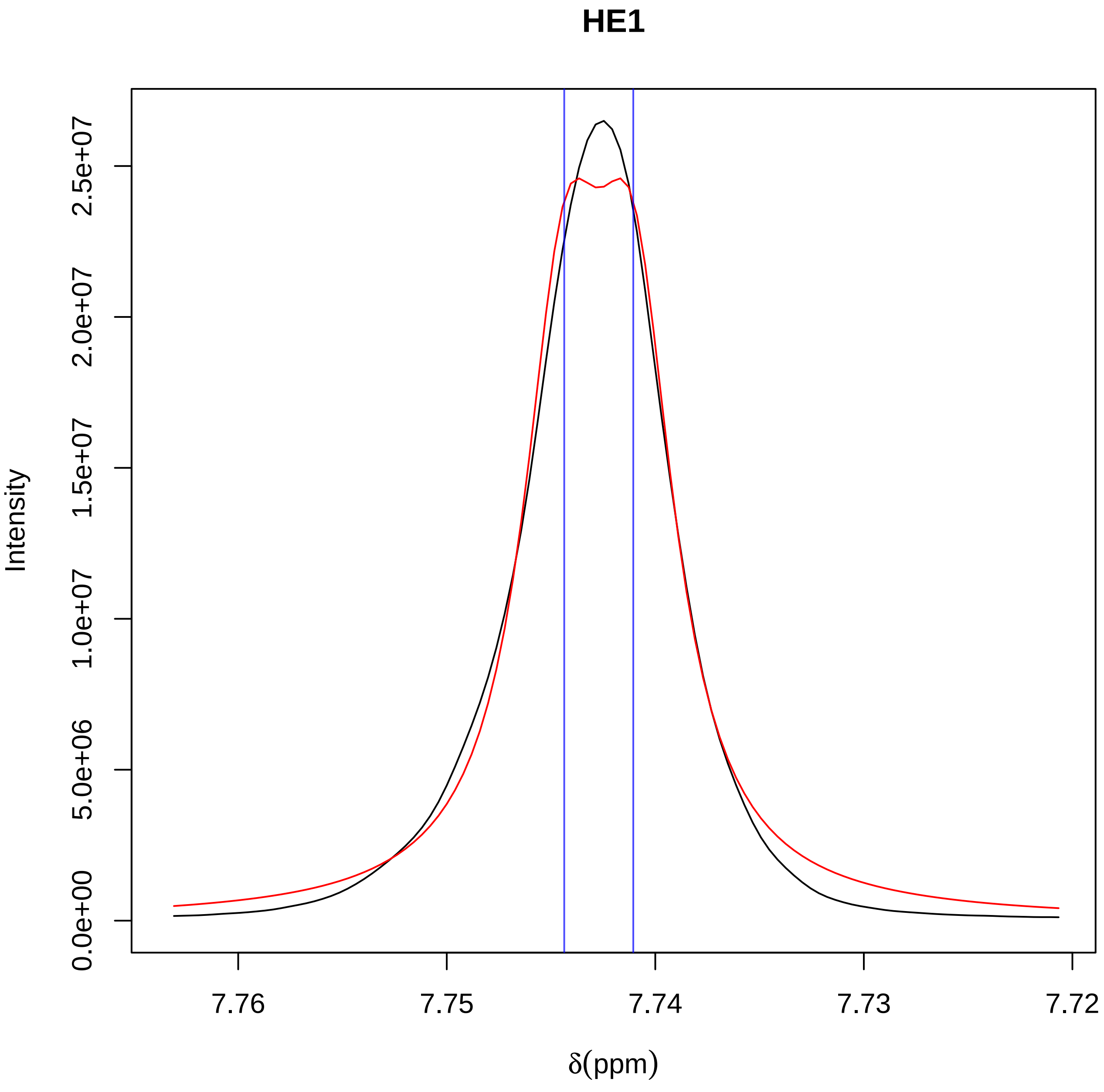

HE1
Intensity
0.0e+00
5.0e+06
1.0e+07
1.5e+07
2.0e+07
2.5e+07
7.76
7.75
7.74
7.73
7.72
δ(ppm)
